# Association of smoking cessation patterns and untreated smoking with glaucoma, cataract, and macular degeneration: a population-based retrospective study

**DOI:** 10.1038/s41598-024-65813-8

**Published:** 2024-06-26

**Authors:** Yuan-Tsung Tseng, Sheng-Tang Huang, Chun-Hsiang Wang, Ling-Yu Wang, Yi-Chun Kuo

**Affiliations:** 1 Department of Medical Research, Tainan Municipal Hospital (Managed by Show Chwan Medical Care Corporation), Tainan, Taiwan; 2 Department of Ophthalmology, Tainan Municipal Hospital (Managed by Show Chwan Medical Care Corporation), Tainan, Taiwan; 3 Department of Optometry, Chung Hwa Medical University, Tainan, Taiwan; 4 Department of Ophthalmology, Ministry of Health and Welfare, Sinying Hospital, No. 73, Xinyi St., Tainan, Taiwan; 5 Department of Public Health, National Cheng Kung University, Tainan, Taiwan; 6 Department of Hepatogastroenterology, Tainan Municipal Hospital (Managed by Show Chwan Medical Care Corporation), Tainan, Taiwan; 7 Department of Ophthalmology, National Cheng Kung University Hospital, College of Medicine, National Cheng Kung University, Tainan, Taiwan; 8 Department of Ophthalmology, Yunlin Christian Hospital, Yunlin, Taiwan

**Keywords:** Eye diseases, Health care

## Abstract

This study aims to assess the association between nicotine replacement therapy (NRT), varenicline, and untreated smoking with the risk of developing eye disorders. We employed a new-user design to investigate the association between NRT use and the incidence of eye disorders by the Taiwan National Health Insurance program. This study included 8416 smokers who received NRT and 8416 smokers who did not receive NRT (control group) matched using propensity scores between 2007 and 2018. After adjustment for relevant factors, a multivariable Cox regression analysis revealed that compared with untreated smokers, NRT use was associated with a significantly reduced risk of macular degeneration (hazard ratio [HR]: 0.34; 95% confidence interval [CI]: 0.13–0.87, P = 0.024). When stratified by dose, short-term NRT use (8–28 defined daily doses) was associated with significantly lower risk of glaucoma (HR: 0.35; 95% CI: 0.16–0.80, P = 0.012) and a trend toward reduced risk of cataract (HR: 0.60; 95% CI: 0.36–1.01, P = 0.053) compared to no treatment. However, these associations were not observed with long-term NRT use. The results of this real-world observational study indicate that NRT use, particularly short-term use, was associated with a lower risk of certain eye disorders compared to no treatment for smoking cessation. Long-term NRT use did not demonstrate the same benefits. Thus, short-term NRT may be a beneficial treatment strategy for reducing the risk of eye disorders in smokers attempting to quit. However, further evidence is required to verify these findings and determine the optimal duration of NRT use.

## Introduction

Nicotine replacement therapy (NRT) and varenicline (Champix) are effective treatments for smoking cessation, which work by reducing a smoker’s craving for nicotine and delaying relapse. These treatments for smoking cessation have been approved for use by the Food and Drug Administration (FDA)^1–3^.

Many studies have confirmed the safety and effectiveness of NRT and varenicline as smoking cessation treatments. Therefore, they have become the most commonly used medication for smoking cessation^4^.

Smoking has a serious impact on ophthalmic diseases such as iritis, blindness, vision loss, cataract, conjunctivitis, and macular degeneration^5,6^. A large cohort study found that smokers had a higher risk of developing glaucoma compared to non-smokers^7^.

The smoke and toxins in cigarettes irritate the eyes and damage health as well as cause brain damage, affecting visual function^8^.

Several studies have examined the influence of smoking on retinal and brain diseases^9–11^. However, few studies have explored the effects of smoking cessation on ophthalmic diseases. Although many studies have confirmed the safety and efficacy of NRT and varenicline^12^, studies on the effects of long-term use of these treatments on ophthalmic diseases are lacking.

Most studies assessing the risks of complications associated with varenicline have been conducted over a short term. Rare side effects of varenicline are primarily emotional agitation, including restlessness, depression, and suicidal thoughts, as well as potential cardiovascular risk^13^. In 2011 and 2014, the FDA recommended monitoring the cardiovascular risks during varenicline use^14–16^. The adverse effects of NRT are generally mild and well tolerated compared with those of varenicline, which makes NRT a potentially safer and more effective option for use in smoking cessation treatment. However, studies examining the long-term benefits of NRT are lacking^17–19^.

In Taiwan, NRT and varenicline have been included in the National Health Insurance program since 2007 as smoking cessation treatment. Therefore, we used data from the National Health Insurance Research Database (NHIRD) to investigate the impact of smoking cessation on common eye disorders.

## Materials and methods

### Data source

We analyzed data from Taiwan's NHIRD, a population-based claims database that covers more than 99% of Taiwan's population. The NHIRD contains comprehensive data on medical services, procedures, and prescription medications from January 1, 2007, to December 31, 2018, with diagnostic codes in accordance with the *International Classification of Disease, Ninth Revision, Clinical Modification* (*ICD-9-CM*) and *International Classification of Disease, Tenth Revision, Clinical Modification* (*ICD-10-CM*). Because the NHIRD consists of encoded secondary data and does not allow for individual identification, the requirement for informed consent was waived in accordance with the Computer-Processed Personal Data Protection Law. The study protocol was approved by the Research Ethics Committee of Show Chwan Memorial Hospital on November 4, 2021 (IRB-No: 1101002). The approval was granted with the understanding that the study utilizes de-identified data from the National Health Insurance Research Database (NHIRD), thereby negating the need for individual informed consent. The ethical review specifically addressed the study’s compliance with privacy and data protection standards, ensuring that all patient information remained confidential and secure. Our research adheres to the ethical guidelines for the use of health insurance database research, focusing on safeguarding participant privacy and the responsible use of data in accordance with national and international regulations. For additional information on ethical clearance, contact the Research Ethics Committee: irb@show.org.tw.

### Study design and study participants

In this historical cohort study, we utilized real-world observational data drawn from the National Health Insurance Research Database (NHIRD) to examine the outcomes of outpatients participating in smoking cessation programs between 2007 and 2018. Employing a new-user design, this approach allowed us to include all patients initiating smoking cessation treatment during the study period, with the aim of minimizing selection biases often associated with observational studies. We meticulously tracked each patient's medical history prior to their enrollment in smoking cessation programs to accurately assess comorbidities and ensure a comprehensive evaluation of baseline health status. The index date was defined as the date of the patient's first prescription for smoking cessation treatment, marking the starting point for follow-up in our analysis. This methodological framework enables a thorough investigation into the impact of smoking cessation on the progression of eye disorders, taking into account the temporal relationship between treatment initiation and health outcomes.

We identified all patients who used varenicline and NRT from the index date to the end of 2018, death, or the development of outcomes. We divided the patients into 2 groups: the treatment group, who received NRT, and the control group, which was further divided into 2 subgroups. The first subgroup included non-NRT smokers who had used varenicline, and the second subgroup included non-NRT smokers who had not attempted smoking cessation. The primary outcome assessed was the incidence of glaucoma, cataract, and macular degeneration in all groups.

To indicate the duration of NRT exposure during the follow-up period, we calculated the total doses for each NRT prescription. The defined daily doses (DDDs) were determined according to the World Health Organization's proposal, with one DDD of NRT set at 30 mg/day^20^.

### Potential confounders

In our study, we applied strict inclusion and exclusion criteria to minimize the influence of potential confounders and ensure the comparability of the study groups. We excluded patients who were less than 20 years of age, as this age group is less likely to develop the eye conditions of interest and may have different risk factors compared to older adults^21^. Furthermore, we excluded patients diagnosed with blindness, low vision, or pseudophakia, as well as those who had received a diagnosis of glaucoma, cataract, or macular degeneration before the index date. These exclusions were made to ensure that the study population was free of pre-existing eye conditions that could bias the results^22^.

We enrolled all users who were matched by exact age, sex, Charlson comorbidity index (CCI), comorbidities (hypertensive cardiovascular disease [HCD], hyperlipidemia, diabetes mellitus^23^, and chronic kidney disease [CKD]), medications (aspirin, statins, angiotensin-converting enzyme inhibitors [ACEIs], β-blockers, and selective serotonin reuptake inhibitors [SSRIs]), and the index date.

### Covariate assessment

In our study, we adjusted for potential confounders based on the association between varenicline and nicotine. Specifically, we adjusted for sex, age, and CCI as well as comorbidities such as HCD (ICD-9 codes 401–405; ICD-10 codes I10–I15), hyperlipidemia (ICD-9 code 272; ICD-10 code E78), DM (ICD-9 code 250; ICD-10 codes E10.0, E10.1, E10.9, E11.0, E11.1, and E11.9), CKD (ICD-9 code 585; ICD-10 code N18), and medication history (including low-dose aspirin, statins, ACEIs, β-blockers, NSAIDs, spironolactone, glucocorticoids, and SSRIs) that may affect eye disease incidence. We also considered the following eye-related outcomes: blindness and low vision (*ICD-9* code 369; *ICD-10* code H54), and pseudophakia (*ICD-9* code 379.31; *ICD-10* code H27.0).

### Main outcome measurements

We determined the presence of the following eye diseases in the first diagnosis: glaucoma (*ICD-9* code 365; *ICD-10* codes H40, H42), cataract (*ICD-9* code 366; *ICD-10* codes H25-H26, H28), and macular degeneration (*ICD-9* codes 362.0–362.5; *ICD-10* codes E11.3, H34–H35).

The diagnoses of glaucoma, cataract, and macular degeneration were established using standardized methods that adhere to internationally recognized criteria, ensuring accuracy and consistency in diagnosis across the study population. For glaucoma, diagnostic methods included visual field assessment using automated perimetry to evaluate visual field defects, evaluation of the optic nerve and retinal nerve fiber layer using ophthalmoscopy and optical coherence tomography (OCT)^24^, and measurement of intraocular pressure (IOP)^25^. It is noteworthy that normal-tension glaucoma, characterized by progressive glaucomatous optic neuropathy despite normal IOP, is prevalent among the Asian population^26^. For cataract, diagnostic methods included slit-lamp examination to assess the degree of lens opacity and visual acuity measurement using the Snellen chart^27^. For macular degeneration, diagnostic methods included fundus examination using ophthalmoscopy or fundus photography and optical coherence tomography (OCT) to evaluate the retinal thickness and morphology^28^.

### Exposure definition and follow‑up

The new-user design included a washout period of at least 2 years to mitigate the impact of external factors on patients with newly diagnosed target outcomes, including Glaucoma, Cataract, and Macular Degeneration^29^.

Patients who did not experience the target outcomes or who died during follow-up were censored. To avoid immortal time bias, we defined the index date as the date of the first prescription for varenicline or nicotine for each user. For each matched comparison group, the exposure period began when NRT was initiated. All patients were followed up from the index date to 2018.

We followed up every participant’s prescriptions and clinical services until December 31, 2018. The follow-up duration was defined as the interval from the index date of both groups to the date of diagnosis of target outcomes, death, or December 31, 2018.

### Statistical methods

Categorical variables were assessed using the McNemar test, whereas paired *t* tests were used to compare some continuous variables of prescription and baseline characteristics. Propensity score matching (PSM) was employed to mitigate potential selection bias by balancing baseline characteristics between the varenicline and nicotine cohorts.

### Propensity score matching

We performed 1:1 full matching without replacement by using the R package “MatchIt” (version 4.3.4) to conduct robust PSM. This allowed the regression model to be specific to the function of the outcome variable on the treatment variable, and addressing extreme weights through trimming was not necessary after PSM^30,31^. We used Cox proportional hazards regression models to calculate the hazard ratios (HRs) and 95% confidence intervals (CIs) and the bootstrapping method to ensure the stability and robustness of our model^32,33^.

The cumulative incidence of the different study cohorts was measured using the Kaplan–Meier method, and the differences in curves were examined using the log-rank test. All statistical analyses were performed using SPSS version 21.0 (SPSS Inc., Chicago, IL, USA) and R version 3.4.3 (R Core Team, 2017). Statistical significance was determined using a 2-tailed test with a *P* value of < 0.05.

### Institutional review board statement

In accordance with the Declaration of Helsinki, the present study involving human subjects received approval from the Institutional Review Board of Show Chwan Memorial Hospital (IRB-No: 1080703).

### Informed consent statement

Considering that the NHIRD dataset consists of encrypted secondary data, making it impossible to identify individuals, the Institutional Review Board at Show Chwan Memorial Hospital in Changhua City, Taiwan, waived the necessity for informed consent on August 30, 2019 (IRB No: 1080703).

## Results

### Patient characteristics

**Figure 1:**
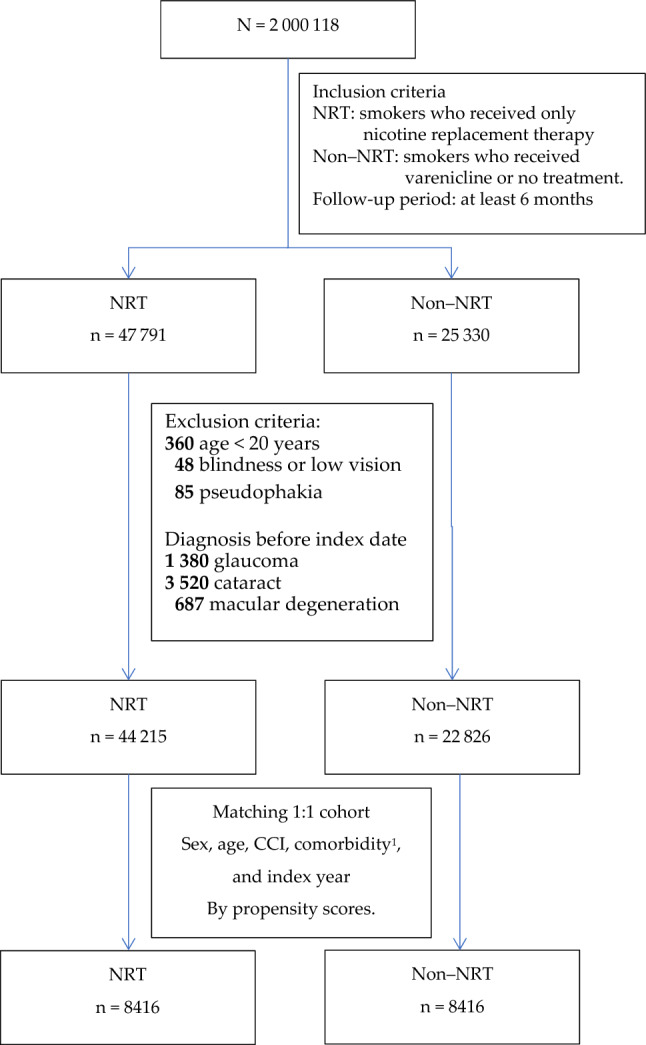
Flowchart of patient selection. Patients were selected based on the presence of comorbidities (*HCD* hypertensive cardiovascular disease; hyperlipidemia, *DM* diabetes mellitus, *CKD* chronic kidney disease), medication use (aspirin; statins; ACEI, angiotensin-converting enzyme inhibitors; β-blockers; and SSRIs, selective serotonin reuptake inhibitors.), and the index year (exact year of the first prescription).

Of the 2,000,118 individuals in our database, 16,832 were sampled as smoking outpatients between 2002 and 2018 by using a new-user design. An at least 2-year washout period before the index date of treatment initiation was included for all patients. Through 1:1 matching by age, sex, CCI, comorbidities (HCD, hyperlipidemia, DM, and CKD), and medications (aspirin, statins, ACEIs, β-blockers, and SSRIs), we identified 8416 smokers who only received NRT as the NRT group and 8416 non-NRT controls (5189 received varenicline alone and 3227 received no treatment for smoking cessation), with the exact year of the first prescription considered as the index date. (Fig. 1).

We conducted a 1:1 matched cohort study without replacement that included baseline demographic, medical, and prescription information, which ensured a well-balanced and robust comparison between the NRT and non-NRT cohorts. The cohorts were matched by sex, age, CCI scores, and follow-up period. The male-to-female ratio was 84.9:15.1 (*P* = 1.000), with a mean age of 42.9 ± 11.9 (*P* = 1.000) and mean CCI value of 0.8 ± 1.3 (*P* = 1.000). The mean follow-up period was 3.39 ± 2.27 years for the NRT group and 3.34 ± 2.27 years for the non-NRT group, with a median follow-up duration of 3.17 years for both groups (Table 1).

**Table 1:** Baseline characteristics of nicotine replacement therapy (NRT) and control groups.

	NRT N = 8416	%	Non–NRT N = 8416	%	P
Sex					
F	1271	15.1	1271	15.1	1.000
M	7145	84.9	7145	84.9	
Age	42.9 ± 11.9		42.9 ± 11.9		1.000
CCI	0.8 ± 1.3		0.8 ± 1.3		1.000
HCD					
N	7442	88.4	7442	88.4	1.000
Y	974	11.6	974	11.6	
Hyperlipidemia					
N	7446	88.5	7446	88.5	1.000
Y	970	11.5	970	11.5	
DM					
N	7929	94.2	7929	94.2	1.000
Y	487	5.8	487	5.8	
CKD					
N	8385	99.6	8385	99.6	1.000
Y	31	0.4	31	0.4	
Aspirin					
N	6983	83.0	6983	83.0	1.000
Y	1433	17.0	1433	17.0	
Statins					
N	7740	92.0	7740	92.0	1.000
Y	676	8.0	676	8.0	
ACEI					
N	7689	91.4	7689	91.4	1.000
Y	727	8.6	727	8.6	
β-Blockers					
N	6021	71.5	6021	71.5	1.000
Y	2395	28.5	2395	28.5	
SSRI					
N	7509	89.2	7509	89.2	1.000
Y	907	10.8	907	10.8	

*NRT* nicotine replacement therapy, *CCI* Charlson Comorbidity Index, *HCD* hypertensive cardiovascular disease, *DM* diabetes mellitus, *CKD* chronic kidney disease, *ACEI* angiotensin-converting enzyme inhibitors, *SSRIs* selective serotonin reuptake inhibitors.

### Comparison of complications between the subgroups of different demographics, comorbidities, and comedications

A multiple Cox regression analysis was conducted with adjustment for various confounders, including age, sex, CCI, comedications (aspirin, statins, ACEIs, β-blockers, and SSRIs), and comorbidities (hypercholesterolemia, hyperlipidemia, DM, and CKD), to evaluate the association of NRT with the risk of progression of glaucoma, cataract, and macular degeneration, and adjusted hazard ratios (aHRs) were calculated.

As shown in Table 2, age was significantly associated with increased risks of glaucoma (adjusted HR: 1.04, 95% CI: 1.01–1.06, *P* = 0.002), cataract (aHR: 1.13, 95% CI: 1.11–1.14, *P* < 0.001), and macular degeneration (aHR: 1.07, 95% CI: 1.03–1.12, *P* < 0.001) in the NRT group compared with non-NRT group. Moreover, hyperlipidemia was significantly associated with the increased risks of glaucoma (aHR: 2.60, CI: 1.18–5.70, *P* = 0.017) and cataract (aHR: 2.46, CI: 1.39–4.35, *P* = 0.002) in the NRT group compared with non-NRT group. No significant associations were observed between other comorbidities and medication use and the risks of glaucoma, cataract, and macular degeneration in the 2 groups at risk, as revealed by multiple Cox regression analysis.

**Table 2:** Factors associated with glaucoma, cataract, and macular degeneration after adjustment for potential confounders in a multiple Cox regression model with propensity score matching (PSM).

	Glaucoma	P	Cataract	P	Macular degeneration	P
Sex	1.80 (0.70–4.65)	0.225	0.84 (0.45–1.58)	0.586	n/a*	0.975
Age	1.04 (1.01–1.06)	**0.002**	1.13 (1.11–1.14)	**0**.**000**	1.07 (1.03–1.12)	**0**.**000**
CCI	0.98 (0.83–1.16)	0.784	1.06 (0.95–1.19)	0.310	0.84 (0.62–1.14)	0.257
HCD	1.49 (0.65–3.44)	0.350	1.22 (0.72–2.07)	0.465	1.19 (0.30–4.74)	0.807
Hyperlipidemia	2.60 (1.18–5.70)	0.017	2.46 (1.39–4.35)	0.002	1.90 (0.50–7.12)	0.344
DM	0.93 (0.43–2.03)	0.852	1.00 (0.60–1.67)	0.991	2.71 (0.76–9.69)	0.125
CKD	2.30 (0.63–8.41)	0.209	1.58 (0.68–3.71)	0.290	1.89 (0.21–17.2)	0.573
Aspirin	1.07 (0.57–2.00)	0.830	0.72 (0.45–1.15)	0.171	3.65 (1.33–10.0)	0.012
Statins	0.91 (0.41–2.05)	0.826	0.96 (0.54–1.70)	0.891	0.50 (0.14–1.81)	0.294
ACEIs	0.75 (0.33–1.69)	0.489	0.56 (0.32–0.95)	0.032	0.72 (0.20–2.65)	0.623
β–Blockers	1.73 (0.97–3.09)	0.063	1.18 (0.77–1.81)	0.459	1.02 (0.35–3.00)	0.964
SSRI	1.35 (0.65–2.82)	0.427	1.13 (0.59–2.16)	0.713	0.67 (0.09–5.14)	0.702

Factors associated with the development of glaucoma, cataract, and macular degeneration were identified using a Cox regression model with PSM. Hazard ratios (HRs) and 95% confidence intervals (CIs) for each factor are presented after adjustment for potential confounders.

*According to the data protection policy of NHIRD, the data on target outcomes with < 3 cases cannot be provided.

*CCI* Charlson Comorbidity Index, *HCD* hypertensive cardiovascular disease, *DM* diabetes mellitus, *CKD* chronic kidney disease, *ACEI* angiotensin-converting enzyme inhibitors, *SSRIs* selective serotonin reuptake inhibitors, *PSM* propensity score matching, *HR* hazard ratio, *CI* confidence interval.

The incidence rates (per 1000 person-years) of glaucoma, cataract, and macular degeneration are presented in Table 3. The incidence rates of glaucoma (1.18 vs. 1.48 vs. 2.17) and cataract (2.51 vs. 3.17 vs. 4.23) were lower in the NRT group than in the varenicline and untreated groups. However, no significant difference in the incidence rates of macular degeneration was noted between the NRT and non-NRT groups.

**Table 3:** Incidence rates of individual eye complications among subgroups (per 1000 person-years).

Outcome	Glaucoma	IR	(95% CI)	Cataract	IR	(95% CI)	Macular degeneration	IR	(95% CI)
Untreated	17	2.17	(1.26–3.48)	33	4.23	(2.92–5.95)	9	1.15	(0.60–2.21)
Varenicline	25	1.48	(0.96–2.19)	52	3.17	(2.36–4.15)	7	0.89	(0.43–1.87)
NRT	33	1.18	(0.81–1.65)	70	2.51	(1.95–3.17)	9	1.15	(0.60–2.21)

Subgroups are defined based on whether smokers were treated with NRT, varenicline, or were untreated.

*IR* incidence rate, *CI* confidence interval, *NRT* Nicotine Replacement Therapy.

Table 4 adopted multivariate Cox regression analysis to estimate the associations between various treatment modalities and the development of glaucoma, cataract, and macular degeneration, as well as the relationship between different doses of NRT and the occurrence of these ocular diseases. Firstly, the analysis found no statistically significant association between the NRT cohort and the occurrence of glaucoma (HR, 0.77; 95% CI, 0.49–1.22, P = 0.267), cataract (HR, 0.76; 95% CI, 0.55–1.04, P = 0.089), or macular degeneration (HR, 0.55; 95% CI, 0.24–1.24, P = 0.148) when compared to the non-NRT cohort. Secondly, when the non-NRT group was further stratified into untreated and varenicline subgroups, a statistically significant difference was observed between the NRT group and the untreated subgroup in terms of macular degeneration (HR, 0.34; 95% CI, 0.13–0.87, P = 0.024).

**Table 4:** Multivariate associations between glaucoma, cataract, and macular degeneration, by treatment.

	Glaucoma	P	Cataract	P	Macular degeneration	P
Non-NRT	1.00		1.00		1.00	
NRT	0.77 (0.49–1.22)	0.267	0.76 (0.55–1.04)	0.089	0.55 (0.24–1.24)	0.148
Untreated	1.00		1.00		1.00	
Varenicline	0.67 (0.36–1.28)	0.226	0.65 (0.28–1.48)	0.303	0.41 (0.14–1.16)	0.094
NRT	0.60 (0.33–1.09)	0.093	0.75 (0.53–1.07)	0.116	0.34 (0.13–0.87)	**0.024**
Untreated	1.00		1.00		1.00	
Varenicline	0.66 (0.35–1.26)	0.209	0.92 (0.58–1.44)	0.702	0.40 (0.14–1.15)	0.089
1–7 DDDs	0.50 (0.20–1.24)	0.135	0.81 (0.46–1.44)	0.476	0.15 (0.02–1.22)	0.075
8–28 DDDs	0.35 (0.16–0.80)	**0.012**	0.60 (0.36–1.01)	0.053	0.39 (0.13–1.20)	0.102
29–56 DDDs	0.91 (0.39–2.13)	0.835	0.72 (0.36–1.47)	0.372	0.48 (0.10–2.25)	0.354
> 56 DDDs	1.23 (0.55–2.76)	0.621	0.95 (0.49–1.84)	0.877	0.29 (0.04–2.26)	0.235

The multivariate analysis investigated the associations between treatments and the development of glaucoma, cataract, and macular degeneration as well as the relationship between different doses of NRT and the occurrence of these eye disorders. Potential confounding factors were controlled for in the analysis and a defined daily dose (DDD) of 30 mg/day of NRT was used. The adjusted hazard ratios (HRs) and 95% confidence intervals for each treatment and dose category are presented. The P-values in bold indicate significance at p < 0.05.

*NRT* nicotine replacement therapy, *DDDs* defined daily doses.

**Figure 2:**
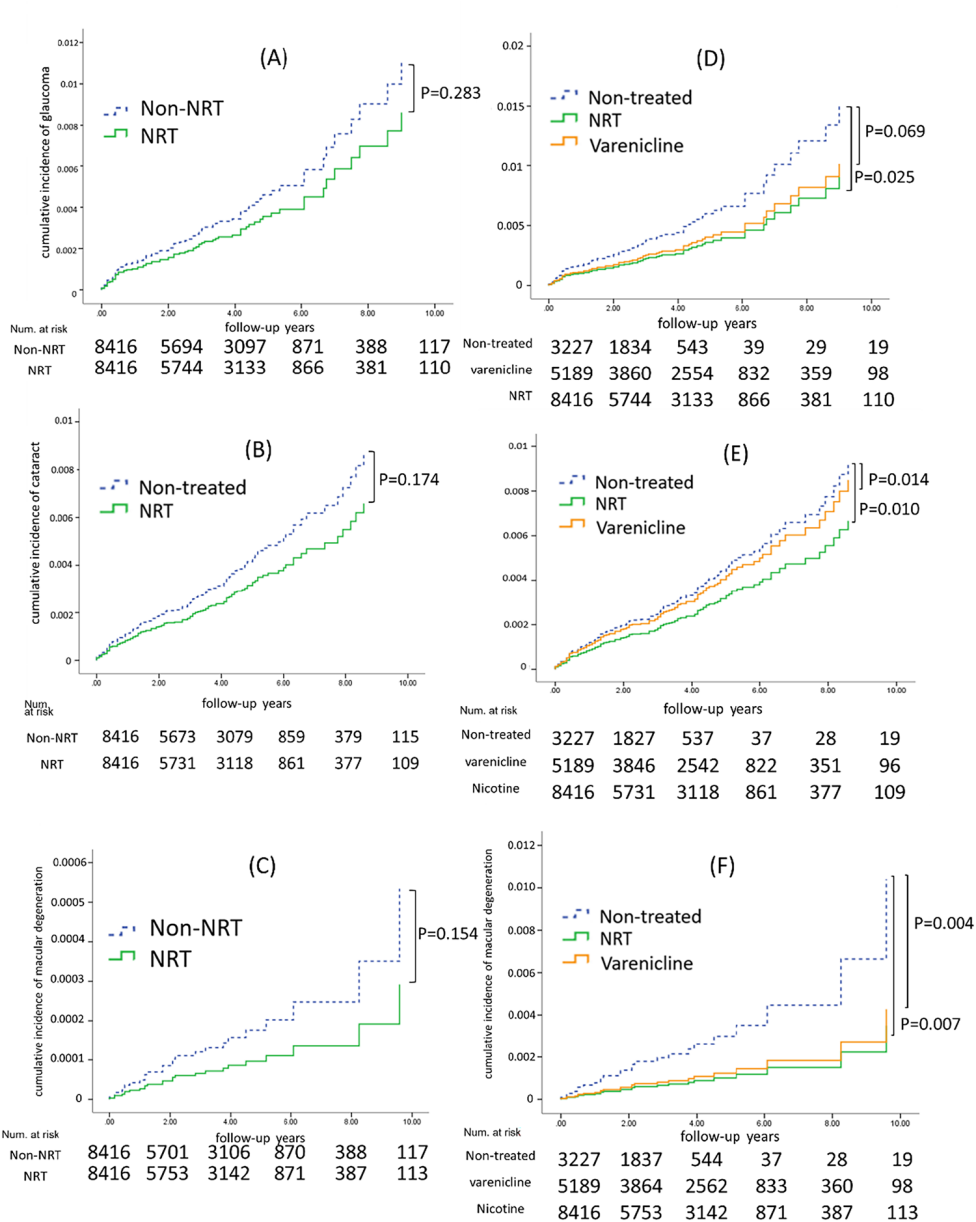
Cumulative incidence of glaucoma, cataract, and macular degeneration among smokers receiving and not receiving nicotine replacement therapy (NRT). Panels (**A**)–(**C**) show the cumulative incidence of each eye complication over time among smokers who received NRT compared with those who did not. Panels (**D**)–(**F**) show the cumulative incidence of each eye complication among smokers who received NRT, varenicline, or no smoking cessation medication. All data in the panels were obtained using the Kaplan–Meier method, and statistical significance was assessed using the log-rank test. *NRT* nicotine replacement therapy.

Furthermore, no significant differences in the risks of glaucoma (HR, 0.67; 95% CI, 0.36–1.28, P = 0.226), cataract (HR, 0.65; 95% CI, 0.28–1.48, P = 0.303), or macular degeneration (HR, 0.41; 95% CI, 0.14–1.16, P = 0.094) were observed between the varenicline and untreated groups. Finally, Kaplan–Meier analysis demonstrated that the NRT group had significantly lower risks of glaucoma (P = 0.025), cataract (P = 0.010), and macular degeneration (P = 0.007) compared to the varenicline or untreated cohort (Fig. 2B,D,F).

### Sensitivity analyses

**Figure 3:**
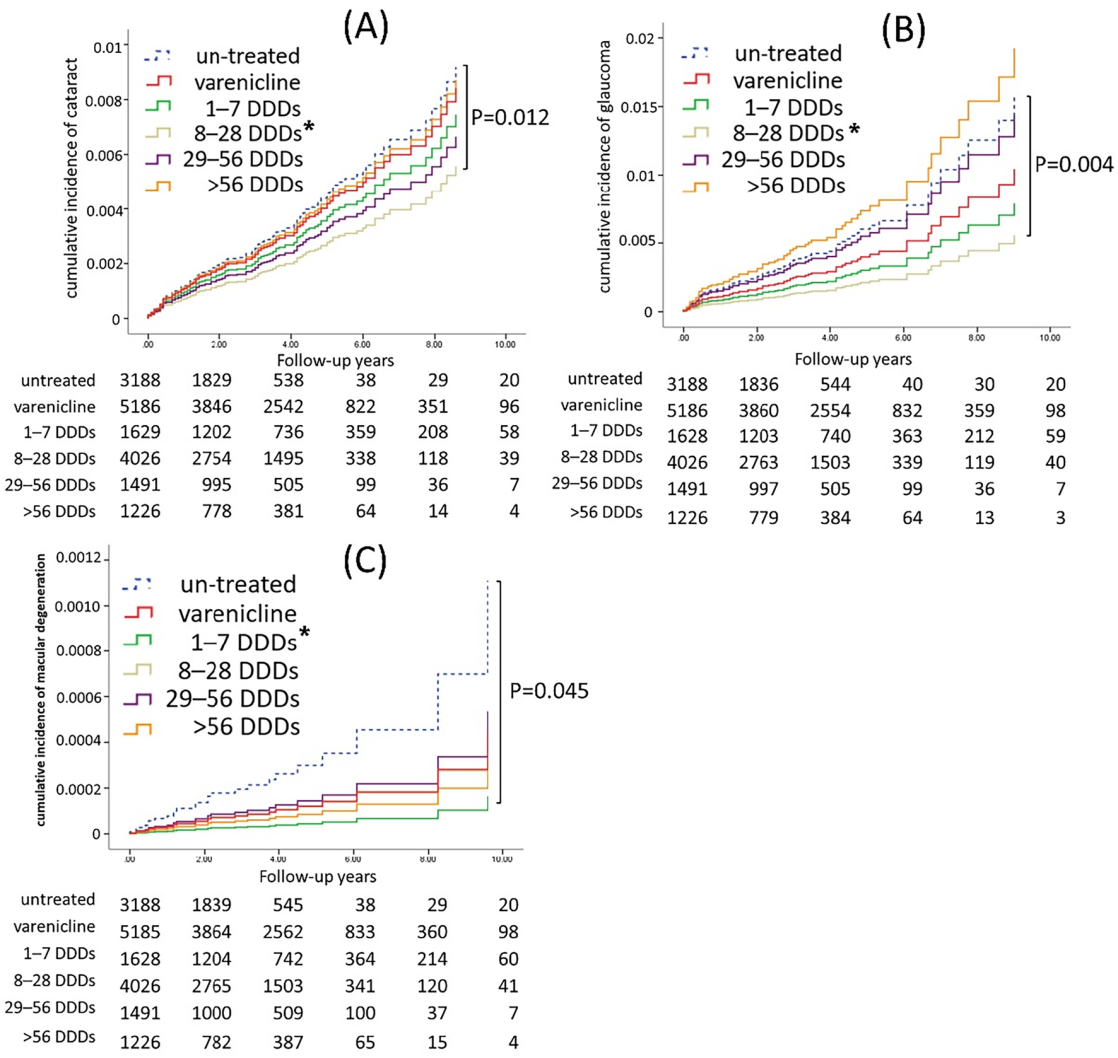
Cumulative incidence of glaucoma (**A**), cataract (**B**), and macular degeneration (**C**) in smokers stratified by an NRT defined daily dose (DDD) of 30 mg/day according to WHO recommendations. The Kaplan–Meier curves were generated, and statistical significance was evaluated using the log-rank test. The study comparing the target outcomes between smokers who used NRT and those who used varenicline or no smoking cessation medication. The DDDs marked with an asterisk (*) indicate statistical significance.

The sensitivity analyses performed in our study suggest that the reduction in the risks of glaucoma (HR, 0.35; 95% CI, 0.16–0.80, P = 0.012) and cataract (HR, 0.60; 95% CI, 0.36–1.01, P = 0.053) was more substantial in patients who used 8–28 DDDs of NRT compared with groups of other DDDs as well as untreated smokers. As shown in Fig. 3, Kaplan–Meier analysis revealed that the 8–28 DDD group had significantly lower risks of glaucoma (P = 0.004) and cataract (P = 0.012) compared with the varenicline or untreated cohort, although no significant association was observed for the risk of macular degeneration. However, a subgroup analysis based on the smoking cessation duration revealed no significant association between the usage of 1–7 DDDs, 8–14 DDDs, or > 56 DDDs and the risks of these eye diseases in smoking patients.

## Discussion

To the best of our knowledge, our study is the first to compare the risks of glaucoma, cataract, and macular degeneration among smokers treated with NRT, treated with varenicline, and untreated smokers for smoking cessation. No study has compared the progression of these eye disorders after smoking cessation among these groups by using a new-user design with balanced baseline characteristics.

### The new-user design for balanced groups

We employed a rigorous 1:1 new-user design with PSM without replacement to compare the outcomes between NRT users and non-NRT smokers (including those using varenicline and untreated smokers) and to adjust for differences in demographics, comorbidities, and comedications^30,31^. To avoid immortal time bias, the participants were assigned to treatment groups based on the date of their first prescription. Multivariate analysis was also conducted to adjust for age, comorbidities, and comedications. To ensure consistency in comorbidity, we used the CCI for balancing the distribution of variables^34^.

### Main results

Our analytical process, as detailed in Table 1, successfully neutralized baseline discrepancies between groups of NRT users and non-users, laying a foundational bedrock for equitable risk analysis pertaining to ocular ailments. Subsequent analyses, encapsulated in Table 2 via multivariate Cox regression following propensity score matching, delineate the comparative risk dynamics for glaucoma, cataract, and macular degeneration across diverse patient demographics, comorbid states, and concurrent medication regimes. A noteworthy revelation from this analysis is the accentuated risk of these eye diseases with advancing age within the NRT cohort, corroborating the well-documented narrative of age as a predominant risk factor^35–37^.

Additionally, our study casts hyperlipidemia into relief as a discernible risk factor for glaucoma and cataract among NRT users, thereby substantiating theories that have previously implicated hyperlipidemia as a potential precipitant for these visual impairments^38,39^. The mechanistic pathways linking hyperlipidemia to heightened ocular disease risk, however, remain to be fully elucidated, underscoring the imperative for continued investigative exploration.

Our investigation also revealed a comparative reduction in the incidence rates of glaucoma, cataract, and macular degeneration among NRT users versus those utilizing varenicline or no treatment at all, as shown in Table 3. Yet, adjustments for confounding variables via multiple Cox regression analyses tempered these observations, revealing no statistically significant correlations between NRT utilization and the onset of the aforementioned eye diseases, as depicted in Table 4 and Fig. 2A–C. Further stratification of non-NRT users into untreated and varenicline-utilizing cohorts, and their subsequent comparison with NRT users, unearthed a markedly diminished risk for glaucoma and cataract among the latter, as depicted in Fig. 2D–F.

The observed lack of a significant link between NRT use and a lowered incidence of ocular conditions might be attributable to the generally low success rates of smoking cessation treatments, where only a minor fraction (4%-7%) of individuals achieve cessation^23,40^. However, despite the low success rate, our study identified an effective prescription pattern for reducing the risk of eye disorders in smokers.

### Differences in complications between the subgroups of different defined daily doses

Our sensitivity analyses, as presented in Table 4 and Fig. 3, shed light on the intricate relationship between nicotine replacement therapy (NRT) dosage and the risk of developing glaucoma, cataract, and macular degeneration among smokers. Notably, we observed a significant reduction in glaucoma risk among smokers who used moderate doses of NRT (8–28 DDDs) compared to untreated smokers, a finding corroborated by Kaplan–Meier analysis (Fig. 3A).

Regarding cataract risk, although the overall use of NRT did not achieve statistical significance compared to the untreated group, subgroup analysis revealed a trend towards reduced risk among the moderate dose (8–28 DDD) group. The Kaplan–Meier curve (Fig. 3B) also reflected this trend, showing a lower cumulative incidence of cataract in the 8–28 DDD group compared to other DDD categories and untreated smokers.

In contrast, while the overall use of NRT was significantly associated with a decreased risk of macular degeneration, dose–response analysis failed to identify a significant association between any specific NRT dosage and the risk of this condition. The Kaplan–Meier curve (Fig. 3C) also did not demonstrate significant differences in the cumulative incidence of macular degeneration across DDD groups.

### Implications of smoking cessation on eye health outcomes

The reasons for this finding could be multifactorial. Successful smoking cessation during regular treatment could contribute to the observed interaction. Alternatively, older patients with certain demographic characteristics, comorbidities, and comedications may smoke a higher number of cigarettes, which could lead to a higher risk of eye complications. However, in our present study, we found no significant risk of eye complications among patients with HCD, DM, and CKD after adjustment for multiple Cox regression. Notably, studies have suggested a potential relationship between smoking cessation and a reduced rate of glaucomatous visual field progression. However, these studies have not compared the durations of smoking cessation among smokers^41–43^.

Our study findings suggest that the protective effects observed from moderate NRT use against glaucoma and the trend towards reduced cataract risk might indicate a potential dose–response relationship. These effects appear to be beneficial in the short term but may not be sustained over the long term. Research has indicated that while NRT may increase smokers' motivation to quit, it does not necessarily lead to a reduction in cigarette consumption. Furthermore, there may not be a linear relationship between NRT usage and the number of cigarettes smoked^44–46^. As a result, long-term NRT treatment may not reduce the risk of eye complications resulting from dependence on smoking cessation.

### Limitations

Our study has limitations in terms of evaluation of the reversal of risk of progression of eye disorders after smoking cessation^47,48^.

First, the NHIRD does not include data on lifestyle factors, such as alcohol consumption and cigarette consumption, and body mass index; as a result, we could not assess the impact of these factors on the risk of the eye disorders. Second, distinguishing the participants’ socioeconomic levels was difficult in our study. Thirdly, the database does not provide information on essential clinical tests, including Best Corrected Visual Acuity (BCVA), which could offer insights into the visual outcomes of our cohort. Fourth, the use of both ICD-9 and ICD-10 classifications for aggregating data on eye diseases. The transition from ICD-9 to ICD-10 occurred during the study period, which may have introduced the possibility of misclassification. Fifth, compliance to smoking cessation medication could not be accurately measured from the NHIRD, which may have affected the estimation of prescription usage. Sixth, information on over-the-counter NRT usage was limited, which may have led to an underestimation of NRT exposure. We suggest that future studies with larger sample sizes and longer follow-up durations should be conducted to further investigate the potential effects of NRT on the risks of glaucoma and cataract.

## Conclusions

This study evidenced the potential association between short-term NRT for smoking cessation and decreased risks of glaucoma and cataract compared with long-term NRT, varenicline, or no treatment. NRT is beneficial in successful smoking cessation, which may be associated with a lower risk of eye disorders. These results may be useful for ophthalmologists and patients who smoke. However, further research is needed to confirm this finding and determine the optimal duration of NRT treatment for obtaining a beneficial effect on the reduction of eye disorder risks in smokers.

## Data Availability

The NHIRD enforces strict confidentiality guidelines to protect personal electronic data. Due to legal restrictions imposed by the Taiwan Ministry of Health and Welfare (MOHW) under the “Personal Information Protection Act,” the results presented in this study are only available from the NHIRD of Taiwan to eligible researchers who meet the criteria for access to confidential data. You can submit a formal proposal for access to NHIRD data by completing the application form and submitting it to the Ministry of Health and Welfare (MOHW). (https://dep.mohw.gov.tw/dos/np-2497-113.html, accessed on 8 August 2023). The contact information for necessary data of the MOHW is as follows: 886-2-85906828; Email: sthuiying@mohw.gov.tw.
